# Radiological diagnosis of prevalent osteoporotic vertebral fracture on radiographs: an interim consensus from a group of experts of the ESSR osteoporosis and metabolism subcommittee

**DOI:** 10.1007/s00256-024-04678-4

**Published:** 2024-04-25

**Authors:** Yì Xiáng J. Wáng, Daniele Diacinti, Maria Pilar Aparisi Gómez, Fernando Ruiz Santiago, Fabio Becce, Alberto Stefano Tagliafico, Mahesh Prakash, Amanda Isaac, Danoob Dalili, James F. Griffith, Giuseppe Guglielmi, Alberto Bazzocchi

**Affiliations:** 1 Department of Imaging and Interventional Radiology, Faculty of Medicine, The Chinese University of Hong Kong, Shatin, New Territories, Hong Kong SAR China; 2 Department of Radiological Sciences, Oncology and Pathology, Sapienza University of Rome, Rome, Italy; 3 Department of Radiology, Auckland City Hospital, Auckland District Health Board, Auckland, New Zealand; 4 Department of Anatomy and Medical Imaging, Faculty of Medical and Health Sciences, The University of Auckland, Auckland, New Zealand; 5 Department of Radiology, IMSKE, Valencia, Spain; 6 Department of Radiology and Physical Medicine, Faculty of Medicine, University of Granada, Granada, Spain; 7 Musculoskeletal Radiology Unit, Hospital Universitario Virgen de Las Nieves, Granada, Spain; 8 Department of Diagnostic and Interventional Radiology, Lausanne University Hospital, University of Lausanne, Lausanne, Switzerland; 9 Department of Radiology, DISSAL, University of Genova, Genoa, Italy; 10 Department of Radiology, Ospedale Policlinico San Martino, Genoa, Italy; 11 Department of Radiodiagnosis and Imaging, Post Graduate Institute of Medical Education and Research (PGIMER), Chandigarh, India; 12 School of Biomedical Engineering and Imaging Sciences, King’s College London, London, UK; 13 Academic Surgical Unit, Southwest London Elective Orthopaedic Centre (SWLEOC), Dorking Road, Epsom, London, UK; 14 Department of Radiology, Epsom and St Hellier University Hospitals NHS Trust, Dorking Road, Epsom, London, UK; 15 Department of Clinical and Experimental Medicine, Foggia University School of Medicine, Foggia, Italy; 16 Radiology Unit, Dimiccoli Teaching Hospital Barletta, Barletta, Italy; 17 Diagnostic and Interventional Radiology, IRCCS Istituto Ortopedico Rizzoli, Bologna, Italy

**Keywords:** Osteoporosis, Vertebral fracture, Radiograph, Vertebral deformity

## Abstract

When a low-energy trauma induces an acute vertebral fracture (VF) with clinical symptoms, a definitive diagnosis of osteoporotic vertebral fracture (OVF) can be made. Beyond that, a “gold” radiographic standard to distinguish osteoporotic from non-osteoporotic VFs does not exist. Fracture-shaped vertebral deformity (FSVD) is defined as a deformity radiographically indistinguishable from vertebral fracture according to the best of the reading radiologist’s knowledge. FSVD is not uncommon among young populations with normal bone strength. FSVD among an older population is called osteoporotic-like vertebral fracture (OLVF) when the FSVD is likely to be associated with compromised bone strength. In more severe grade deformities or when a vertebra is collapsed, OVF diagnosis can be made with a relatively high degree of certainty by experienced readers. In “milder” cases, OVF is often diagnosed based on a high probability rather than an absolute diagnosis. After excluding known mimickers, singular vertebral wedging in older women is statistically most likely an OLVF. For older women, three non-adjacent minimal grade OLVF (< 20% height loss), one minimal grade OLVF and one mild OLVF (20–25% height loss), or one OLVF with ≥ 25% height loss, meet the diagnosis of osteoporosis. For older men, a single OLVF with < 40% height loss may be insufficient to suggest the subject is osteoporotic. Common OLVF differential diagnoses include X-ray projection artifacts and scoliosis, acquired and developmental short vertebrae, osteoarthritic wedging, oncological deformities, deformity due to high-energy trauma VF, lateral hyperosteogeny of a vertebral body, Cupid’s bow, and expansive endplate, among others.

**Supplementary Information:**

The online version contains supplementary material available at 10.1007/s00256-024-04678-4.

## Introduction

The assessment of osteoporotic vertebral fracture (OVF) “status” provides relevant bone strength information. The future fracture risk increases significantly with the increasing severity of vertebral fracture status [1–6]. However, to date, there is no consensus on the radiographic diagnostic criteria of OVF [7–15]. In this article, we propose an interim consensus for the radiological diagnosis of prevalent OVF on radiographs. The focus is on prevalent OVF in older women and men, rather than on traumatic vertebral fracture (VF) occurring in osteoporotic patients. It is noted that radiographic incident OVF can be defined in various ways depending on the purpose of the studies [10, 15].

With the purpose of creating a more accurate nomenclature, we introduce the concepts of fracture-shaped vertebral deformity (FSVD) and osteoporotic-like vertebral fracture (OLVF) [16, 17]. FSVD is defined as a vertebral deformity that is radiographically indistinguishable from other vertebral fractures. Radiographic FSVD among older populations has been commonly assumed as OVF in literature, which implies the subject suffers from osteoporosis. However, the diagnosis of osteoporosis requires meeting certain criteria [18, 19]. While a VF occurring in a low-energy traumatic setting with associated clinical symptoms would satisfy the criteria for osteoporosis, the detection of certain radiographic FSVDs does not meet the criteria, particularly for male subjects [10, 11, 20]. FSVD among older populations is termed OLVF when we consider the FSVD is likely associated with compromised bone strength. Some radiographic OLVF or OVF may never have subsequent clinical manifestations though they are associated with lower bone strength, they should be classified as an “imaging biomarker” for bone quality. Incident radiographic OLVF or OVF has been used as a “surrogate clinical endpoint” for anti-osteoporosis drug clinical trials [21–23], with effective medication lowering the incidence of OLVF during the study observation period.

## “Background noise” vertebral deformities for OVF assessment

The concept that “physiological wedging” and “degenerative wedging” are not OVF has been well recognized [14, 24–28]. These non-osteoporotic vertebral wedgings are much more common in men than in women [16, 26]. Recently, it was suggested that the non-osteoporotic vertebral wedgings commonly observed among young and middle-aged subjects are due to micro-fracture, without clinical manifestation [16, 29].

FSVD is common both among young and older adults considered to have normal bone strength. In one study [16], FSVD had a prevalence of 8.3% among 21- to 34-year-old Chinese women and a prevalence of 26.0% among 21- to 34-year-old Chinese men, though most of them had a vertebral height loss of < 20%. Among populations between 34 and 44 years of age, a few cases had FSVD with vertebral height loss of ≥ 20% or with endplate depression [16]. FSVD prevalence is higher among subjects with a manual labor history [29]. Even for subjects with normal bone strength, the prevalence of micro-fracture associated FSVD increases with aging.

At the age of around 74 years, acquired short vertebrae (SVa) had a prevalence of around 10% [16, 29–31]. Around this age, osteoarthritic (OA) wedging has a prevalence of approximately 6% among Caucasian women, but OA wedging prevalence may be very low among East Asians [16, 30]. The prevalences of SVa and OA wedging are expected to be low among young populations.

Developmental (congenital) short vertebra (SVd) is not common with a prevalence estimated at around 0.5% [16].

## OLVF diagnosis

**Fig. 1:**
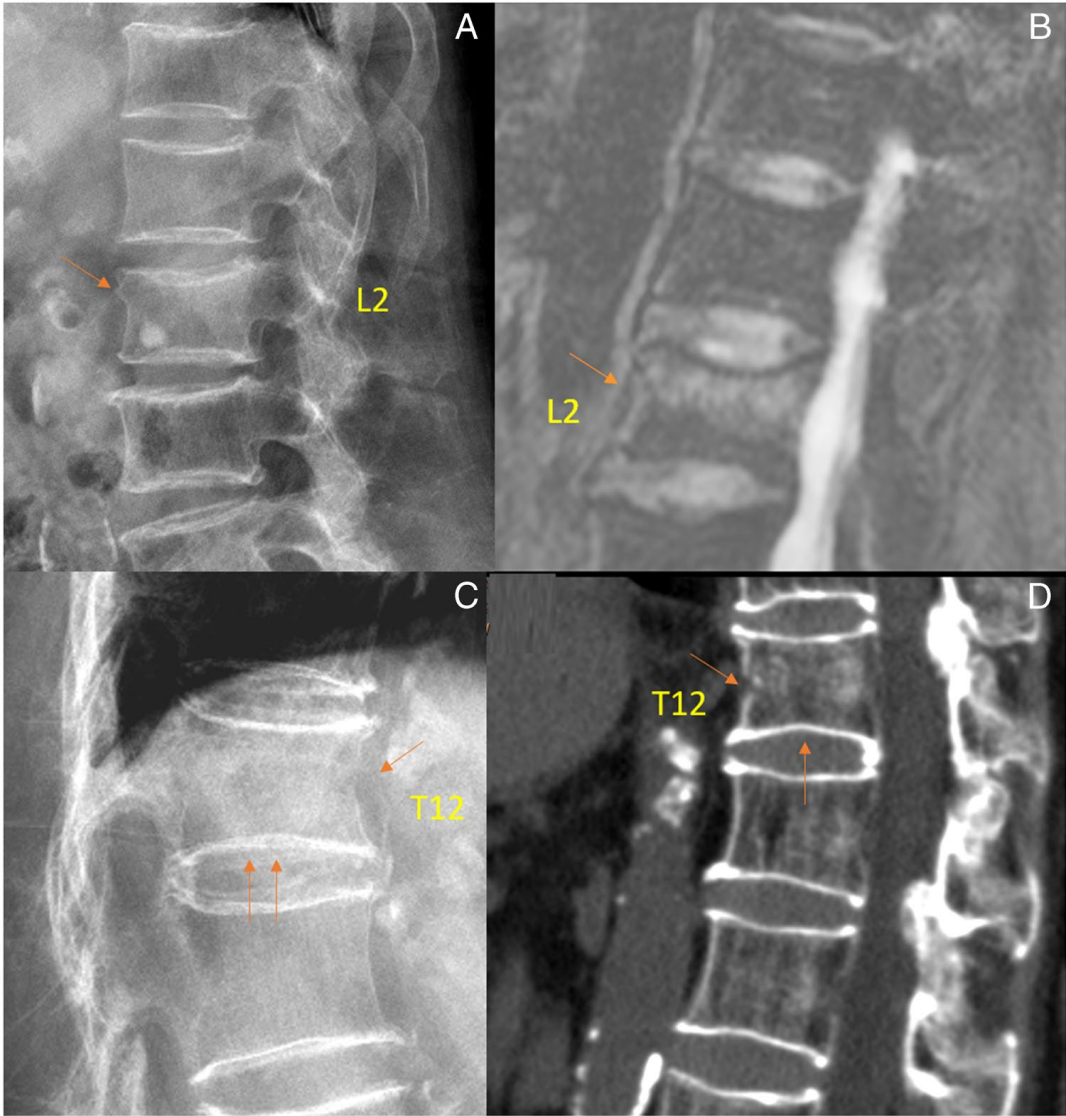
Spine imaging of an 88-year-old woman (**A**, **B**) and a 90-year-old woman (**C**, **D**). Both had a recent low-energy trauma history. For the 88-year-old woman, lateral radiograph (**A**) shows upper endplate depression of L2, with anterior cortex buckling, and slight vertebral height loss. Magnetic resonance imaging (**B**, T2-weighted fat suppressed image) shows L2 endplate depression and bone marrow edema. For the 90-year-old woman, lateral radiograph (**C**) shows indentation of the anterior cortex of T12 (arrow) and lower endplate depression (double arrow). CT shows a fracture of the anterior cortex of T12 (arrow) and slight inferior endplate depression (arrow). Note both involved vertebrae (L2 and T12) have a limited extent of height loss (< 20%). Reproduced with permission from Du and Wáng. Osteoporos Int. 2022;33:1569–1577

When the occurrence of low-energy trauma induces an acute VF with clinical manifestations, a definite diagnosis of OVF can be made (Fig. 1; Suppl Fig. 1) [8, 9]. Beyond that, a “gold” radiographic standard to separate osteoporotic from non-osteoporotic VFs in every case does not exist [32–35]. VF and their repair/healing can occur in the absence of any appreciable radiographic change in vertebral shape [36]. For severe grade deformities or collapsed vertebrae, OVF diagnosis can be made with a relatively high degree of certainty. In mild vertebral deformities, OLVF is often diagnosed based on a high probability rather than being a diagnosis of certainty [8, 11, 29, 37, 38]. This probability depends on many factors, including the sex and age of the subjects and also the physical activity history of the subjects.

**Fig. 2:**
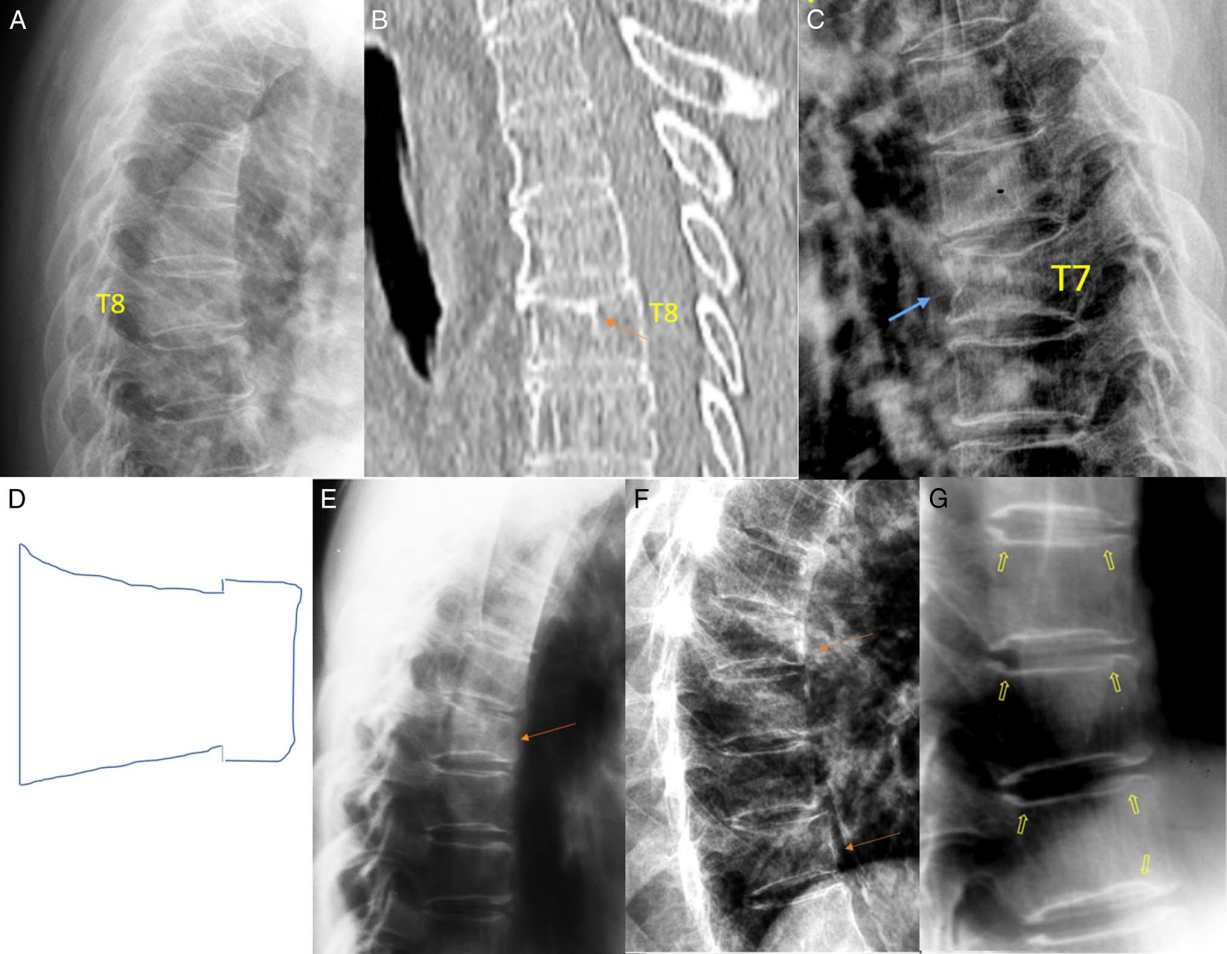
Different OLVF shapes. Radiograph (**A**) shows minimal wedging of T8, without definite upper endplate depression. CT (**B**, same patient as in **A**) shows loss of height of T8 and apparent superior endplate depression (arrow). **C** a typical bi-concave OLVF. In addition to its typical location in the mid-thoracic region, both the superior and inferior endplates are depressed, and anterior cortex indentation is also noted (arrow). **D**, **E**, and **F**, a common OLVF shape which appears as what could be called “mixed-shape,” which is a combination of anterior wedging, bi-concavity, and “stair-step” appearances. The anterior height is reduced but to a lesser extent than the mid portion of the vertebra. **D** Schematic drawing, **E** and **F**, three OLVF showing “mixed-shape” (arrows). **G** a normal segment of thoracic spine shows “stair-step” appearance (arrows). OLVF, osteoporotic-like vertebral fracture. **A **and **B **reproduced with permission from Du and Wáng. Osteoporos Int. 2022;33:1569–1577. **C**, **D**, **E**, **F**, and **G **Reproduced with permission from Wáng. Quant Imaging Med Surg. 2022;12:3495–3514

Radiographic FSVD is diagnosed based on vertebral morphology. Genant et al. [14] noted that “aside from morphometric features, most vertebral fractures are readily distinguished by the presence of endplate deformities and buckling of cortices, by the lack of parallelism of end plates, and by the loss of vertical continuity of vertebral morphology.” Typical OLVFs are bi-concave with anterior wedging. Atypical OVFs can have various shapes and OLVF can also appear as simple wedging without radiographic endplate depression (Fig. 2; Suppl Fig. 2). After excluding known mimickers, singular vertebral wedging in older women is statistically most likely an OLVF, and OLVF is the most common FSVD among older women [17, 37, 38].

Endplate depression (endplate fracture) is a common manifestation of OLVF [34, 39], and the existence of endplate depression as a sign can increase the confidence for OLVF diagnosis. CT is more sensitive for endplate depressions than radiography (Fig. 2A) [38]. However, endplate depression is not an essential sign of OLVF [29], and endplate depression can be occasionally seen among subjects with normal bone strength [16]. Moreover, radiographic OLVF with < 20% vertebral height loss and without endplate depression among older women has also been shown to be associated with lower BMD and a higher incidence of VF during follow-up [17, 29].

Although morphometric methods are intended to be quantitative [40, 41], point placement remains subjective. Pure morphometric methods can lead to falsely classifying degenerative wedging as OLVF [42, 43]. On the other hand, true OLVFs that do not meet the morphometric thresholds may be missed [42]. The International Society of Clinical Densitometry does not recommend vertebral morphometry alone for diagnosis but recognizes morphometry is useful for the evaluation of fracture severity and follow-up [10, 44].

During follow-up, OLVF in older subjects can also repair and recover [11, 17].

In the case of X-rays taken in children, pediatric vertebral development changes should not be considered as deformity [45, 46].

## OLVF grading

Microscopic trabecular fracture and repair, vertebral wedging, endplate and/or cortex fracture (ECF), and vertebral crushing are a spectrum of presentations of compromised vertebral bone strength [8], thus any grading criteria will be subjective. In the early 1990s, Genant and colleagues proposed a semi-quantitative (SQ) grading scheme to evaluate OVF [14]. This grading has become the most used criteria among the radiological community. According to Genant’s SQ criterion, each vertebral body from T4 to L4 is classified as normal (grade 0), mild (grade 1, approximately 20–25% depression in height and a reduction in area 10–20%), moderate (grade 2, approximately 25–40% depression in height and a reduction in area 20–40%), or severe (grade 3, more than 40% reduction in height and area) fracture. OLVFs that do not meet the threshold for fracture are classified as grade 0.5. The criterion for area reduction has been dropped over the years as it is difficult to visually estimate the percentage of area loss. Note that the SQ criteria also stress the importance of qualitative/radiological evaluation [14].

Genant’s SQ criteria may work well for daily clinical practice, but they may cause problems when it comes to recording research results [32, 35, 37]. Genant et al. described OLVFs that do not meet the threshold for fracture as SQ grade 0.5. However, this can cause confusion as to whether SQ grade 0.5 should be considered as an OLVF [8, 9]. SQ method relies on the visual estimation of vertebral dimensions for grading, which is a source for disagreement.

Wang et al. proposed an extended version of the semi-quantitative (eSQ) criteria [47]: (1) minimal grade refers to radiological OLVF with < 20% height loss; (2) mild grade is the same as Genant SQ mild grade (≥ 20 ~ 25% height loss); (3) SQ moderate grade is divided into two subgrades ≥ 25% ~ 33% height loss and ≥ 33% ~ 40% height loss; (4) SQ severe grade is divided into two subgrades ≥ 40% ~ 67% height loss and ≥ 67% height loss (collapsed grade) (Table 1). To avoid inconsistency of vertebral height loss estimation by different readers, eSQ advocates evaluation of vertebral height loss by measurement, with the heights of neighboring normal-appearing vertebrae as references [47]. There could be considerations as to whether a minimal vertebral loss threshold, such as 5 or 10%, should be adopted. However, not only such a threshold will be artificial; any kind of threshold will also involve measurement inconsistency and subjectivity.

**Table 1:** Vertebral height loss criteria for OLVF Genant semi-quantitative (SQ) grading and extended semi-quantitative gradings (eSQ)

Extent of vertebral height loss^¶^						
Grading*^,^ ^§^	< 20%	≥ 20%–25%	≥ 25%–33%	≥ 33%–40%	≥ 40%–67%	≥ 6 7%
Genant SQ^#^	Grade-0.5	Mild	Moderate		Severe	
Extended SQ (eSQ)^#^	Minimal	Mild	Moderate	Moderately-severe	Severe	Collapsed
OLVF score	− 0.5	− 1	− 1.5	− 2	− 2.5	− 3

*OLVF* osteoporotic-like vertebral fracture

^*^ Vertebrae with normal radiographical morphology is noted as grade-0 and OLVF score = 0. ^#^The grading is based on the extent of vertebral height loss, which is not suitable for fracture at acute or subacute phase. This is particularly the case for Genant SQ, as many clinically relevant vertebral fractures show 20% vertebral height loss at acute phase

^§^For experienced readers, there is usually a very good agreement on the “yes” or “no” of the existence of a vertebral deformity (except for those with very minimal deformity). However, disagreement on the grading is common and can only be resolved by measuring with an agreed method

^¶^Visual estimation has a strong inclination to over-estimate the extent of vertebral height loss, and it is advisable to prepare a reference image database for readers

**Fig. 3:**
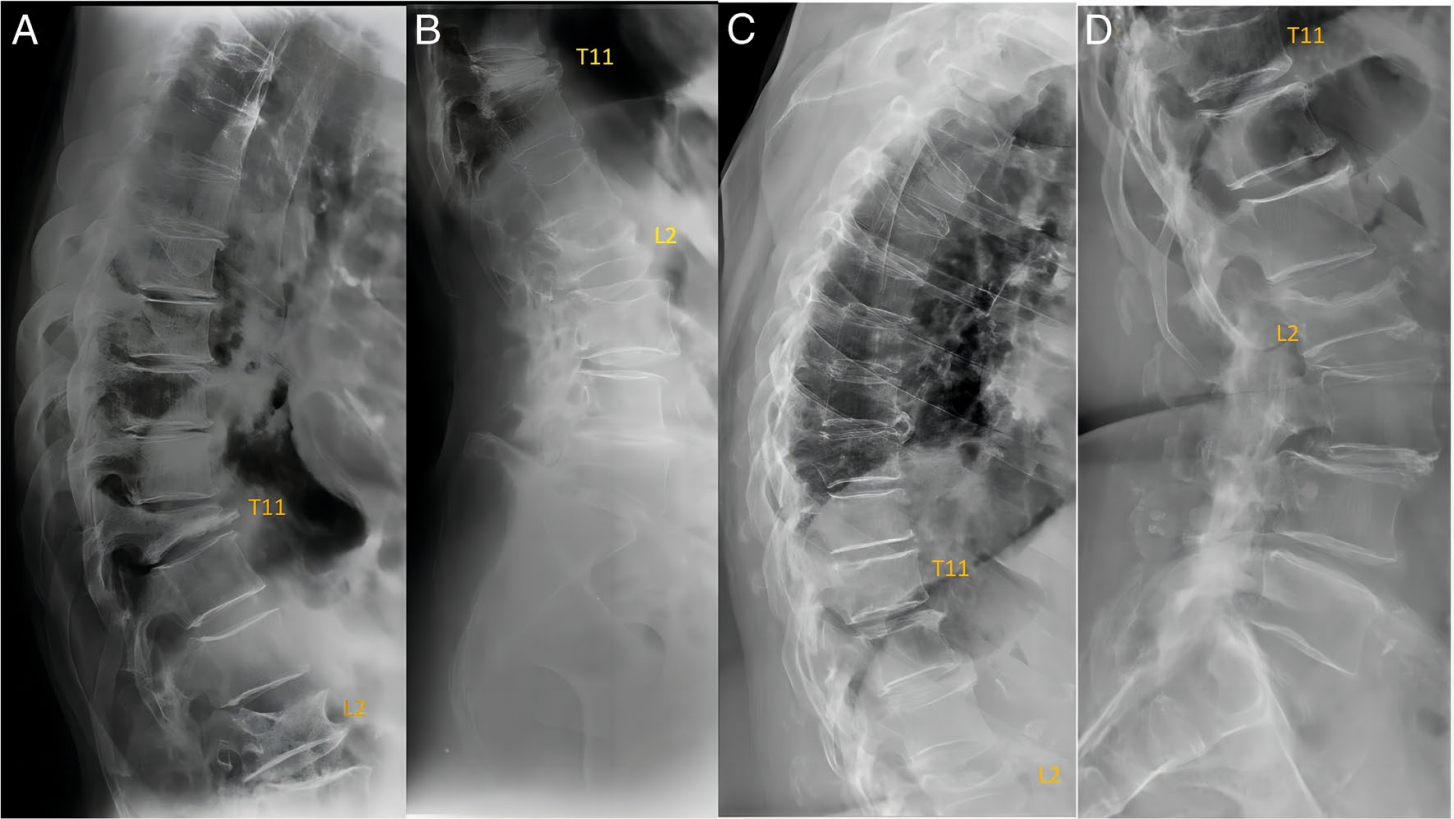
The X-ray projection has important implications in the appearance of vertebrae. **A** and **B** are from an elderly woman, and (**C**) and (**D**) are from a different elderly woman. **A** and **B** show vertebra collapse of T11. Note the extent of height loss at L2 appears to be more severe in **B** than in **A**. It is likely that L2 is more “off-center” with respect to the X-ray beam focus in (**B**) than that in (**A**). The morphology of the superior endplate of T11 mimics a depression on **D**, while on (**C**) the superior endplate of T11 appears to be normal. Note the extent of height loss at L2 appears to be more severe in (**C**) than in (**D**). It is likely that L2 is more “off-center” with respect to the X-ray beam focus in (**C**) than in (**D**). Reproduced with permission from Wáng. Quant Imaging Med Surg. 2022;12:3495–3514

The height loss estimation and thus the grading of an OVF may depend on how “off-center” this vertebra is to the X-ray beam focus (Fig. 3). Therefore, to grade or even diagnose OLVF for vertebrae at the peripheral regions of a radiograph, or for the vertebrae with apparent “bean-can” appearance of the endplates, great care should be taken. For formal OVF evaluation, usually a thoracic spine lateral radiograph is taken, with the X-ray beam focused on T8 (or T7), and a lumbar spine lateral radiograph is taken with the X-ray beam focused on L3 (or L2). Ideally, a third thoracolumbar junction lateral radiograph is additionally taken with the X-ray beam focusing on T12. However, in practice and particularly for opportunistic OVF detection, the X-ray beam focusing is often “off-center” with respect to the OVF.

It is noted that SQ, eSQ, and morphometric measurement criteria are not intended for traumatic OVF classification. For traumatic OVF classification in acute or subacute phases, different criteria such as the AO Spine Thoracolumbar Injury Classification and Severity score (AOSpine-TLICS) and the German Society for Orthopaedics and Trauma (DGOU) classifications are more appropriate [48–50].

## Assessing the severity of osteoporosis based on OLVFss

According to the WHO criteria, the prevalence of femoral neck BMD densiometric osteoporosis is defined the same as the lifetime risk of hip fragility fracture (about 16% for Caucasian women) [18]. If the lifetime risks of fragility fracture at the spine, hip, or forearm are considered, the prevalence of all-inclusive BMD densiometric osteoporosis is approximately 30% for Caucasian women. Efforts have been made to define what portion of older community women and men with a particular severity of radiographic OLVF correspond to a particular T-score status [51, 52]. For each vertebra in a woman, according to the eSQ scheme, a score of 0, − 0.5, − 1, − 1.5, − 2, − 2.5, and − 3 is assigned for no OLVF or OLVF of < 20%, ≥ 20% ~ 25%, ≥ 25% ~ 33%, ≥ 33% ~ 40%, ≥ 40%–67%, and ≥ 67% vertebral height loss, respectively (Table 1). Two adjacent minimal OLVFs are assigned as − 0.5, and three adjacent minimal OLVFs are assigned to be − 1 [29]. The OLVF sum score (OLVFss) is calculated by summing up the scores of vertebrae T1 to L5 (OLVF at T1–T3 levels are rare, and T1–T3 are ignored if not always well shown on radiographs). Conceptually, OLVF sum score (OLVFss) correlates with the severity of osteoporosis (or with no osteoporosis).

For older women, the results of Wang et al. [51] suggested that, statistically, OLVFss ≤  − 1 meets the T-score_lowest_ (the lowest of femoral neck, total hip, and lumbar spine T-scores) ≤  − 2.5 criteria for suggesting osteoporosis. OLVFss ≤  − 1.5 meets the T-score_neck_ (femoral neck T-score) criteria for diagnosing osteoporosis. Following the same principle, for older Chinese men, Wang et al. [52] described OLVFss ≤  − 2.5 suggests the subject is osteoporotic according to T-score_lowest_, and OLVF ≤  − 3 meets osteoporosis diagnosis criteria according to T-score_neck_. According to the work of Wang et al., a single OLVF of 33–40% height loss is insufficient to diagnose a patient as being osteoporotic [20, 52]. It should be understood that this does not necessarily suggest a moderate grade OLVF in men is not due to osteoporosis. A moderate grade OLVF is still more likely to occur in an osteoporotic man than in a man with normal BMD, but only a single moderate grade OLVF in a man does not itself diagnose this subject as being osteoporotic.

Note that, without prior training, visual estimation is likely to overestimate the extent of vertebral height loss [47]. We advocate that targeted training is offered to spine radiograph readers so that vertebral height losses of < 20%, ≥ 20–25%, and ≥ 25% can be estimated with acceptable reliability.

The results of Wang et al. [51, 52] agree with the “baseline FSVD noise” profile of subjects assumed with normal bone strength [16]. These results also agree with the principle that osteoporosis pharmacotherapy should be considered for women with a recent OVF, higher grade OVF, or multiple fractures. A SQ grade 1, solitary, asymptomatic, incidentally discovered vertebral fracture is of questionable clinical significance [53]. We recommend more validation studies to confirm the studies of Wang et al. [51, 52].

It is possible that rural populations from farming communities may demonstrate a higher prevalence of non-osteoporotic FSVD due to their usual manual labor activities.

## OLVF differential diagnosis

### Oncological deformities

Oncological vertebral deformities and vertebral deformities due to hematological diseases represent the most important differential diagnosis for OLVF. Radiographs sometimes may show some specific features of oncological deformities [34, 54], but to make an accurate diagnosis, further imaging such as MRI is often required (Suppl Fig. 3, Suppl Fig. 4) [34]. A detailed discussion on the differentiation of OLVF from oncological deformities is beyond the scope of this article.

### X-ray projection artifacts

**Fig. 4:**
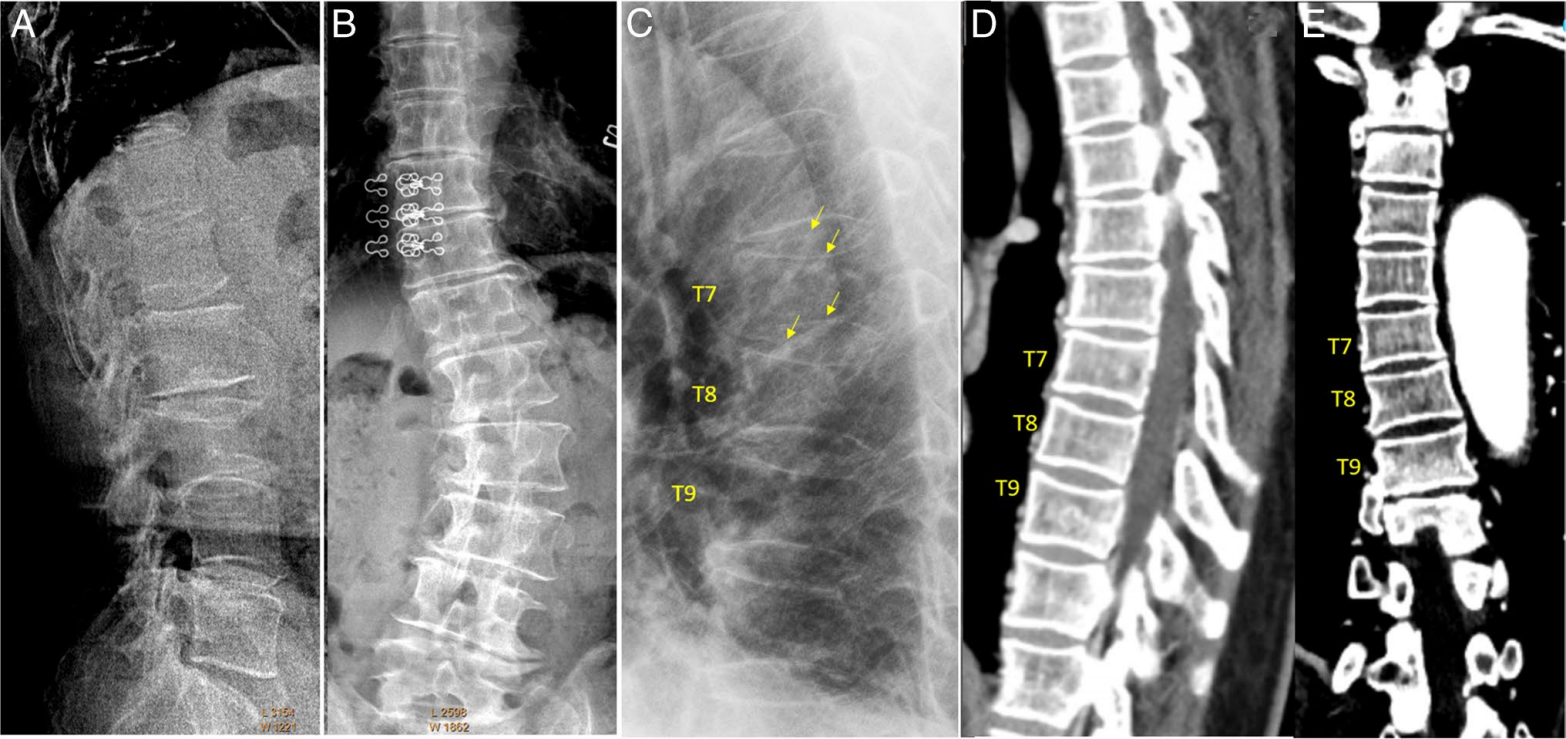
Scoliosis induced artifacts on spine lateral radiographs. These artifacts commonly affect a few adjacent vertebrae. **A** and **B** A case of lumbar spine scoliosis in an elderly woman. Lateral radiograph shows a mimicker of OLVF at L3 with “bean-can” appearances of the endplates. Frontal radiograph demonstrates lumbar spine scoliosis, however lumbar vertebrae appear to be of normal shape. **C**, **D**, and (**E**) Chest imaging of an elderly woman with thoracic spine scoliosis. Lateral radiograph (**C**) shows superior and inferior endplate “depressions” of T7, T8, and T9. Careful observation shows the endplate rings are projected as apparent ovals (“bean-can” appearance) suggesting “rotation” of the vertebrae relative to the expected position (or relative to X-ray beam). Reconstructed sagittal CT image (**D**) and coronal CT image (**E**) demonstrate slight dextroconvex thoracic scoliosis, involving the T6 to T9 vertebrae. There is no vertebral deformity demonstrated for the T7 to T9 vertebrae on images (**D**) and (**E**). Note the anterior vertebral heights of T7 to T9 appear maintained on (**C**). OLVF, osteoporotic-like vertebral fracture. **A **and **B **Reproduced with permission from Wáng. Quant Imaging Med Surg. 2022;12:3495–3514. **C**, **D**, and **E **Reproduced with permission from Du and Wáng. Osteoporos Int. 2022;33:1569–1577

Scoliosis and oblique projections are among the most common causes of diagnostic confusion for OLVF. With these artifacts, the endplate rings project as ovals with a “bean-can” appearance (Fig. 4**)**. At the thoracic and thoracolumbar regions, OLVF is usually associated with some extent of anterior vertebral height loss. For the thoracic and thoracolumbar regions, deformities without any anterior height loss are likely to be due to projection artifacts. However, in the lumbar region, endplate depression can be commonly seen without anterior vertebral height loss (Suppl Fig. 5).

### SVa

**Fig. 5:**
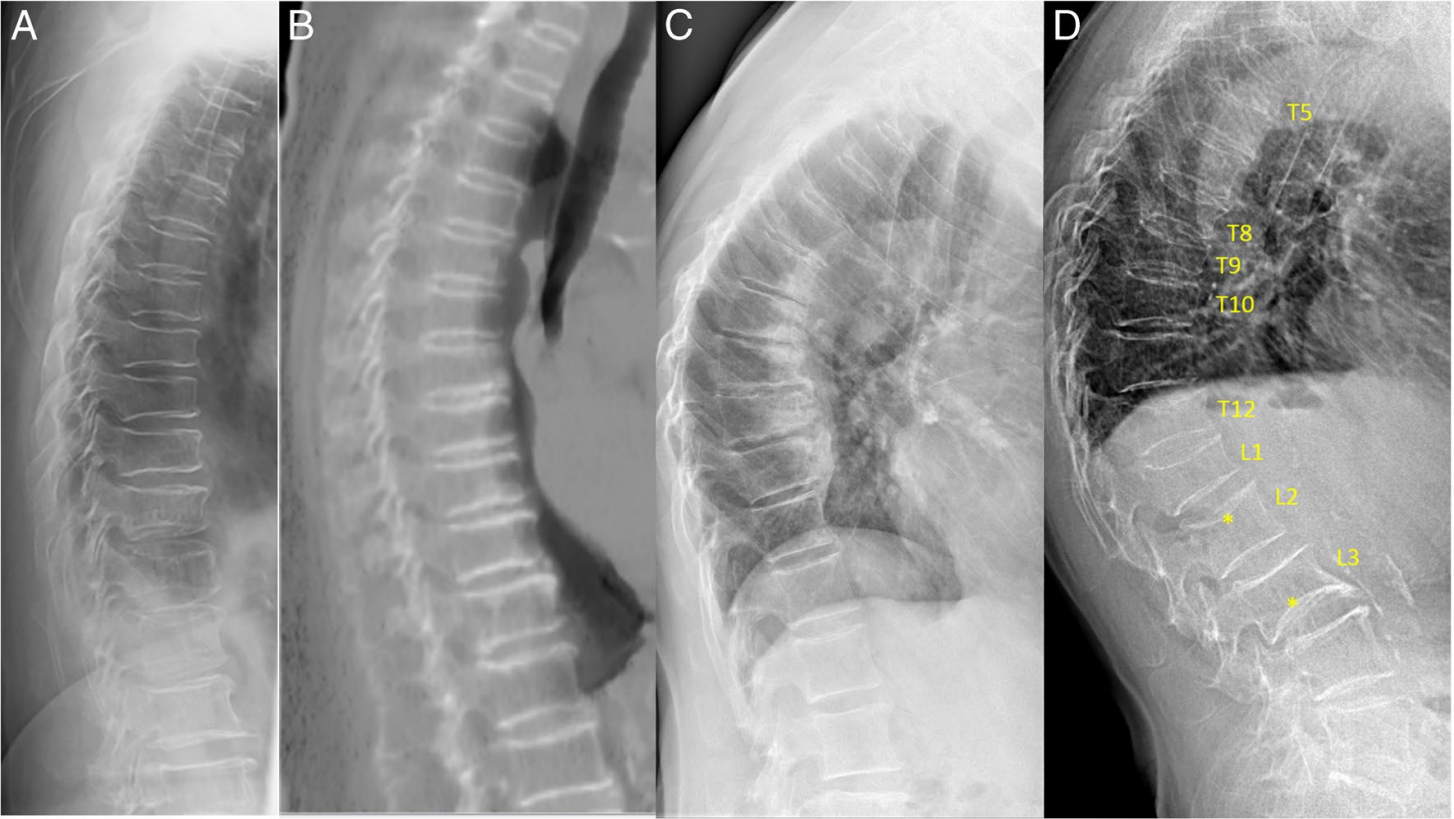
Differentiation of multiple SVa, multiple osteoarthritic (OA) wedging, and multiple OLVFs. **A** Lateral radiograph in a female shows multiple SVa. **B** Sagittal CT reconstruction on another female patient shows multiple SVa. **C** Lateral radiograph demonstrating multiple-level OA wedging in a female patient. **D** Lateral radiograph in a female patient demonstrates multiple OLVFs. In **A** and **B**, the multiple SVa show much less variation in shape and severity, and there is no wedging or apparent endplate depression. In **B**, the increased density of the involved endplates suggests regenerative inflammatory changes. While the many SVa in (**A**) and (**B**) are already quite severe, there is no apparent fracture. OA wedging in (**C**) involves multiple vertebrae at the mid-thoracic spine, and these vertebrae show a similar degree of wedging, with osteophytes. In **D**, OLVFs vary greatly in shape and severity, and most of them show endplate depression. There is a superior endplate fracture (depression) in L2 (asterisk); L3 demonstrates an expansive inferior endplate (anomaly) (asterisk). SVa, acquired short vertebrae; OLVF, multiple osteoporotic-like vertebral fracture. Reproduced with permission from Wáng. Quant Imaging Med Surg. 2023;13:1264–1285

The current consensus defines acquired short vertebrae (SVa) as those in which anterior and middle vertebral heights are decreased to a similar extent but without apparent anterior wedging or bi-concave changes. To diagnose SVa, at least two adjacent short vertebrae in the same subject are required (Fig. 5) [31, 37, 38]. SVa prevalence is associated with aging and manual labor history, and men appear to have a slightly higher prevalence than women [29, 31]. The difference between multiple adjacent SVa and multiple adjacent OLVFs is that multiple adjacent SVa appear similar in appearance, while multiple adjacent OLVFs commonly have different shapes and different severity. Figure 5 also demonstrates that even when many SVa are already quite severe, they do not look fractured in shape.

Developmental short vertebra (SVd) often demonstrates associated changes in the adjacent vertebrae and can be easily diagnosed by an experienced reader (Suppl Fig. 6).

### OA wedging

Osteoarthritic (OA) wedging typically appears as anterior wedging and involves at least two adjacent vertebrae, with similar appearances. OA wedging does not show apparent endplate fracture and does not lead to an increased VF risk on itself [28].

OA wedgings often affect multiple adjacent vertebrae, which appear similarly deformed, while OLVFs tend to affect a single level, with a distinctive loss of the expected vertebral shape (Fig. 5) (Suppl Figs. 7, 8).

While OA wedgings usually demonstrate co-existing disc space narrowing and osteophytes, SVa is more often encountered without associated osteophyte formation [31]. However, SVa and OA wedging share a number of similar features. Sometimes OA wedging and OVF can occur in the same vertebra.

### **SN**

**Fig. 6:**
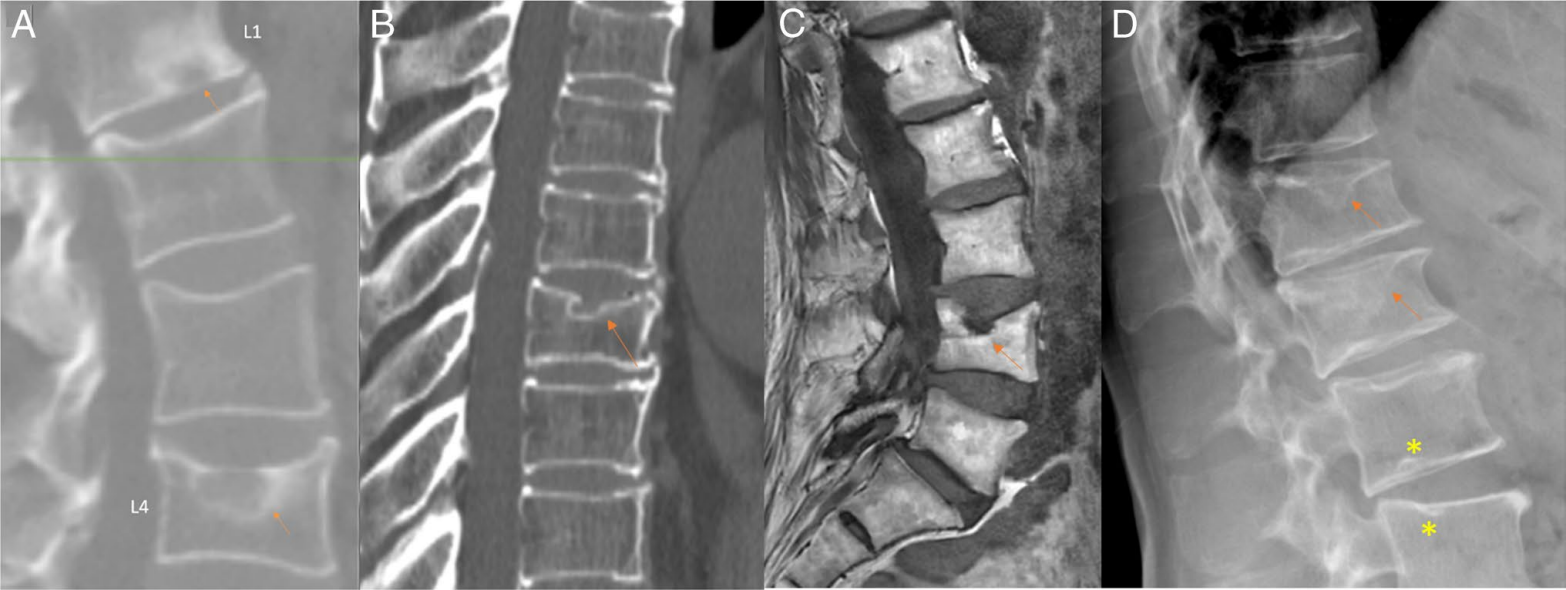
Image examples of Schmorl’s nodes of acquired cause (SNa). **A** Lateral radiograph. There are reactive bone changes in the inferior endplate of L1 (arrow: SNa), and a SNa in the superior endplate of L4 with an associated depression. **B** Reconstructed CT image. The arrow indicates the superior endplate SNa. **C** On T1-weighted sagittal MR image, the L4 OLVF with apparent superior endplate depression and the SNa are demonstrated. **D** Lateral radiograph. There is a superior endplate SNa in L1 together with OLVF, as well as a superior endplate SNa in L2 with apparent endplate fracture. The asterisks in L4 and L5 mark Schmorl’s nodes of developmental cause. OLVF, osteoporotic-like vertebral fracture. Reproduced with permission from Wáng. Quant Imaging Med Surg 2023;13:4044–4049

Schmorl’s node (SN) refers to nucleus pulposus herniation into the vertebral spongy bone. Wang [55] classified SNs into due to primarily developmental cause (SNd) and due to primarily acquired cause (SNa) (Fig. 6; Suppl Fig. 9). SNas are commonly associated with endplate depression. Osteopenic/osteoporotic SN may be a precursor of OVF, a specific type of endplate fracture or a co-phenomenon for advanced OVF [55, 56].

### Deformity due to high-energy trauma VF

Previous high-energy trauma-induced deformity is not rare but less common than OVF mimickers due to scoliosis, projection, SVa, or OA wedging. A portion of OLVFs may be similar in appearance to traumatic VF when a distinct low-energy trauma event had occurred, and anterior cortex fracture is commonly seen in these cases at acute phase (Fig. 1, Suppl Fig. 1**)** [57]. VF due to trauma can demonstrate a fracture of the posterior vertebral wall even in milder grades [58], while “spontaneous” OVF does not. Previous high-energy trauma-induced deformity is expected to involve osteophyte formation.

### Other OLVF mimickers

Many other OLVF mimickers, such as lateral hyperosteogeny of the vertebral body, Cupid’s bow, expansive endplate (Fig. 5D), and Calvé’s disease (eosinophilic granuloma) have been described and extensively illustrated [34, 37, 39]. These changes can be relatively easily differentiated from OLVF by an experienced reader.

**Fig. 7:**
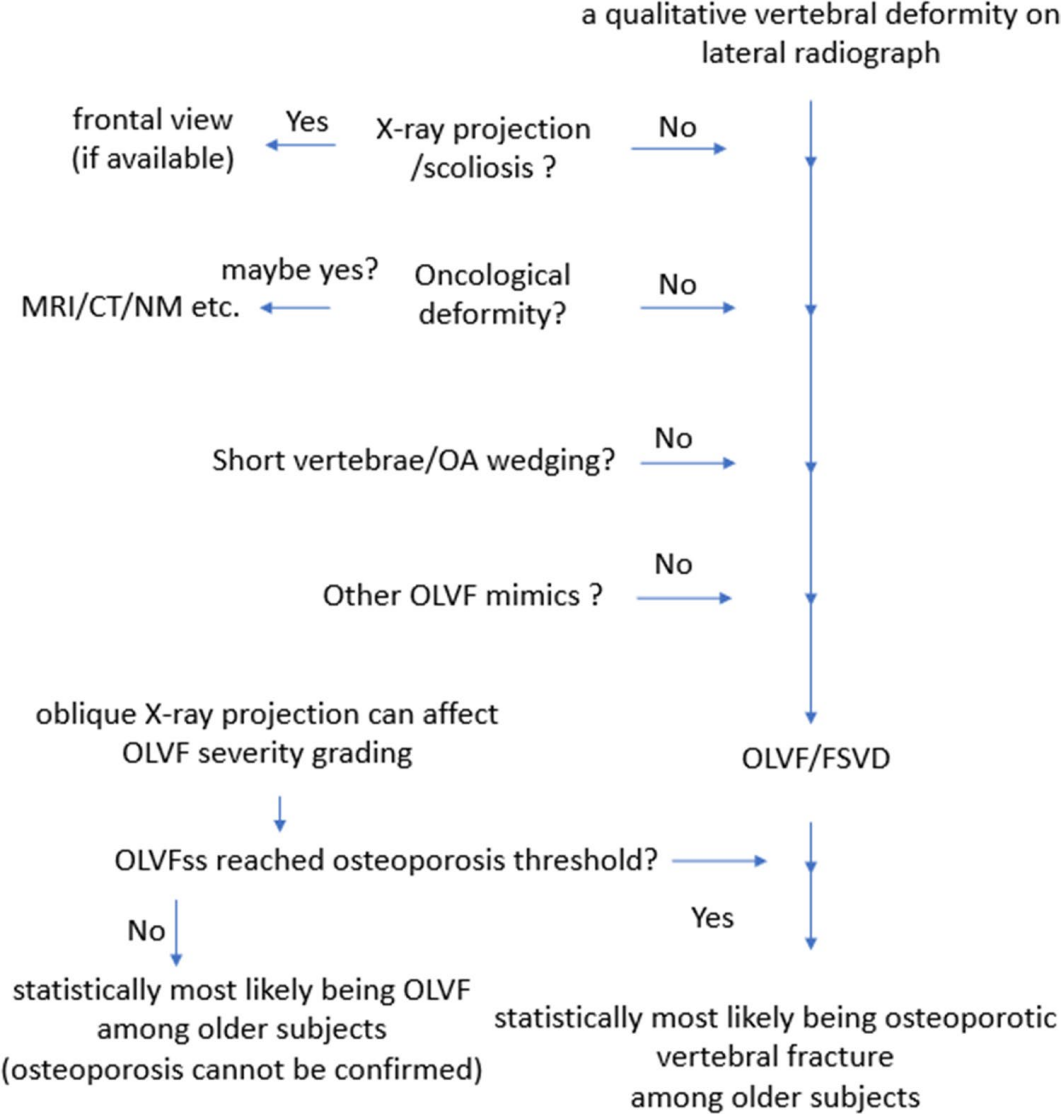
Suggested algorithm of qualitative vertebral deformity evaluation on spine lateral radiograph. At the end of the evaluation, an “OVF” is diagnosed if the OLVFss osteoporosis threshold is reached, or “OLVF” is diagnosed if the OLVFss osteoporosis threshold is not reached [51, 52]. X-ray projection artifacts and scoliosis can be differentiated from OLVF by an experienced reader; however, whether these affected vertebrae also have OLVF cannot be reliably assessed. Both for radiographic OVF and bone mineral density T-score, the diagnosis is based on statistical classification and probability. For patients with densitometrical osteoporosis but without OLVFss criteria for osteoporosis, the detected OLVF can be diagnosed as OVF or remain labeled as OLVF (as it cannot be ascertained if these OLVF are caused by osteoporosis per se). For patients without densitometrical osteoporosis in whom OLVFss criteria for osteoporosis are met, the detected OLVF is diagnosed as OVF

An algorithm for OVF diagnosis is shown in Fig. 7.

## Role of frontal view radiograph for opportunistic OLVF assessment

**Fig. 8:**
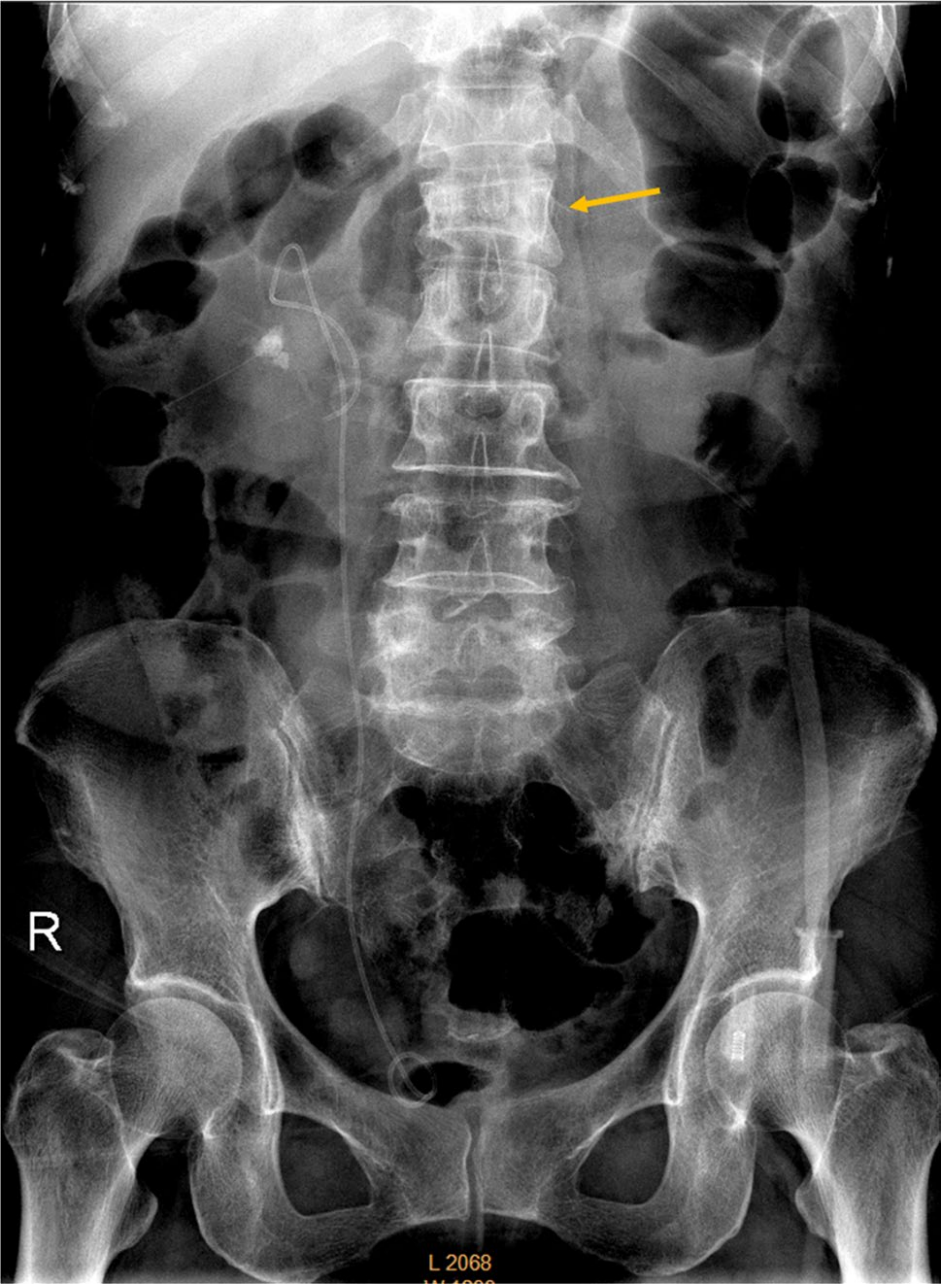
Abdominal radiograph of a 70-year-old female patient. A renal stone and double J catheter are noted on the right side. The arrow indicates an apparent L1 osteoporotic-like vertebral fracture. *Reproduced with permission from Du *et al*. J Orthop Translat. 2021;28:169–178*

Moderate to severe OLVFs at the mid-thoracic, lower thoracic spine, and lumbar spine are mostly identifiable on frontal view spinal, chest, or abdominal radiographs (Fig. 8; Suppl Fig. 10), with a small proportion of ambiguous cases being further clarified by additional lateral views [59]. The frontal radiograph helps to detect artifacts due to scoliosis and oblique X-ray beam projection (Fig. 4).

## Increasing OLVF assessment standardization in epidemiological research to allow inter-study comparison

The prevalence of OVF among older populations is expected to follow the following patterns: (1) as age increases, the prevalence of OVF increases exponentially, both for men and women; (2) the location where OVF prevalence is the highest is the thoracolumbar junction [60, 61]. Note that traumatic VF also has the highest prevalence at the thoracolumbar junction; and (3) the prevalences of both radiographic OVF and clinical OVF in men are no more than half of those in women (Suppl Fig. 11) [20, 62].

The previously reported OVF prevalences tend to allow limited opportunity for inter-study comparison as the inter-reader agreements are not sufficiently good across different reports [10, 37, 63]. The results of morphometric methods also heavily depend on where the measurement cursor is placed [10]. Due to the existence of the uncinate process (or posterior lipping) in some vertebrae, the posterior height measure can cause a high degree of inconsistency. For research purposes, we recommend that the grading of vertebral height loss for SQ or eSQ is measurement-based with a clearly documented methodology to allow inter-study comparison.

In conclusion, except for the scenario in which a low-energy trauma induces an acute VF with clinical symptoms, a “gold” radiographic standard to distinguish osteoporotic from non-osteoporotic VFs does not exist. We favor the use of the term OLVF for prevalent fracture-like deformities, as based on imaging appearances, it is not always possible to diagnose with certainty a VF being of osteoporotic cause. In more severe grade deformities or when a vertebra is collapsed, OVF diagnosis can be made with a relatively high degree of certainty by experienced readers. In “milder” cases, OVF is often diagnosed based on a high probability rather than being an absolute diagnosis. After excluding known mimickers, a singular vertebral wedging in older women is statistically most likely to represent an OLVF [17, 37, 38]. For older women, OLVFss ≤ 1.5 meets the diagnosis of osteoporosis. OVF is much less common among men than among women, and stricter criteria should be applied in diagnosing OVF among men. For older men, a single OLVF with < 40% height loss is insufficient to suggest the subject is osteoporotic. We advocate that targeted training is offered to spine radiograph readers so that OLVF mimickers can be routinely differentiated from OLVF, and vertebral height losses of < 20%, ≥ 20–25%, and ≥ 25% can be estimated with acceptable reliability. For research purposes, we recommend that the grading of vertebral height loss is measurement-based with a clearly documented methodology.

## Supplementary Information

Below is the link to the electronic supplementary material.

Supplementary file1 (DOCX 3538 KB)
