# Supplementary material for: Radiological diagnosis of prevalent osteoporotic vertebral fracture on radiographs: an interim consensus from a group of experts of the ESSR osteoporosis and metabolism subcommittee
Source: Skeletal Radiol. 2024 Apr 25;53(12):2563–74. doi: 10.1007/s00256-024-04678-4 (PMC11493813; doi:10.1007/s00256-024-04678-4)
Supplement: Supplementary file 1 — Supplementary file1 (DOCX 3538 KB) [file 256_2024_4678_MOESM1_ESM.docx]

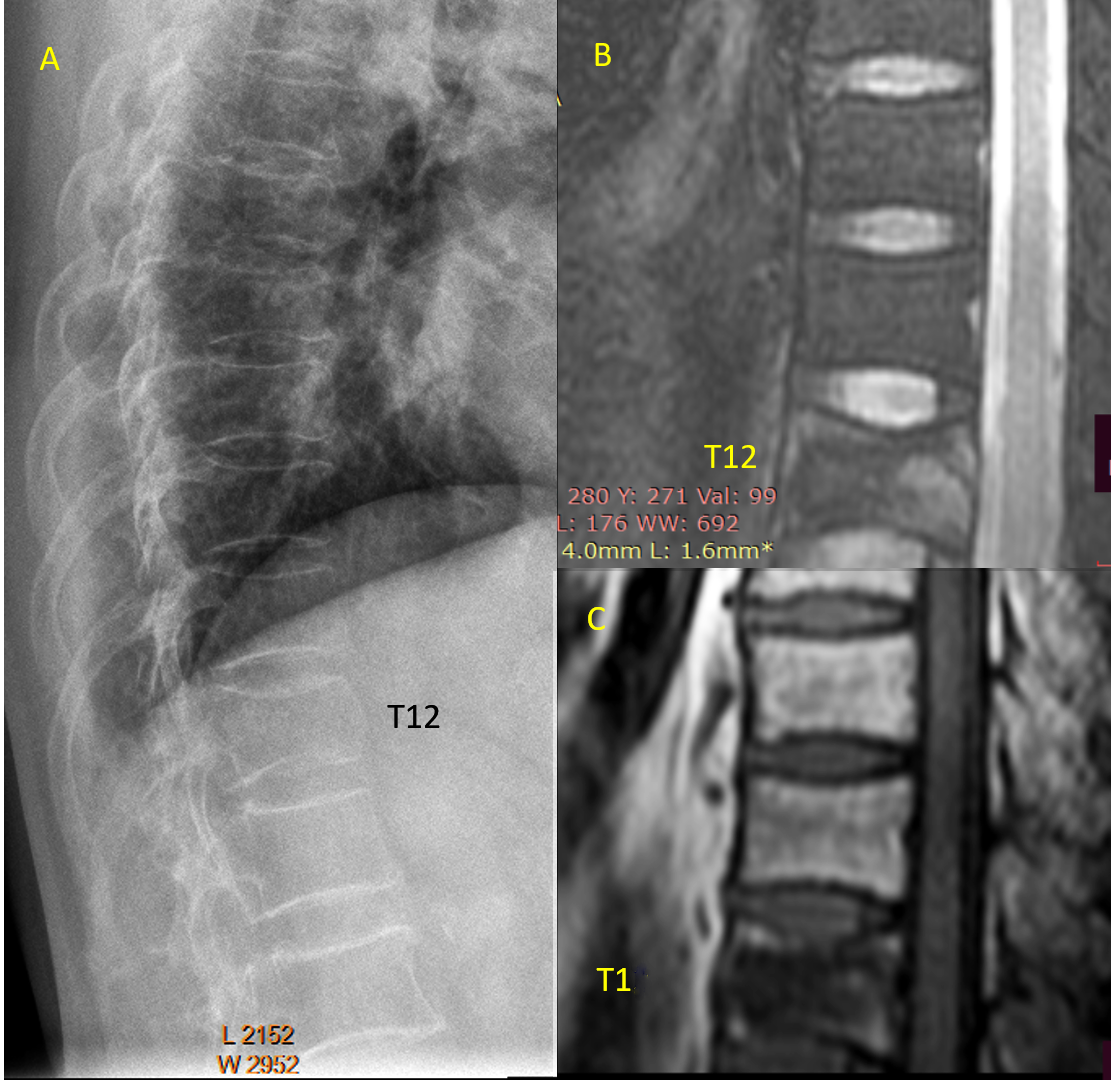


***Suppl Fig-1***. A 67-year-old woman with low energy trauma. Lateral radiograph (**A**) shows T12 deformity, with superior and inferior endplate depression, as well as anterior cortex bulging (swelling). The vertebra shows mixed high signal on fat suppressed T2 weighted MR image (**B**) and mixed low signal on T1 weighted MR image (**C**). Acute osteoporotic vertebral fracture associated with trauma incident commonly show anterior cortex bulging, fracture, or buckling. *Reproduced with permission from* *Wáng. Quant Imaging Med Surg. 2022;12:3495-3514.*


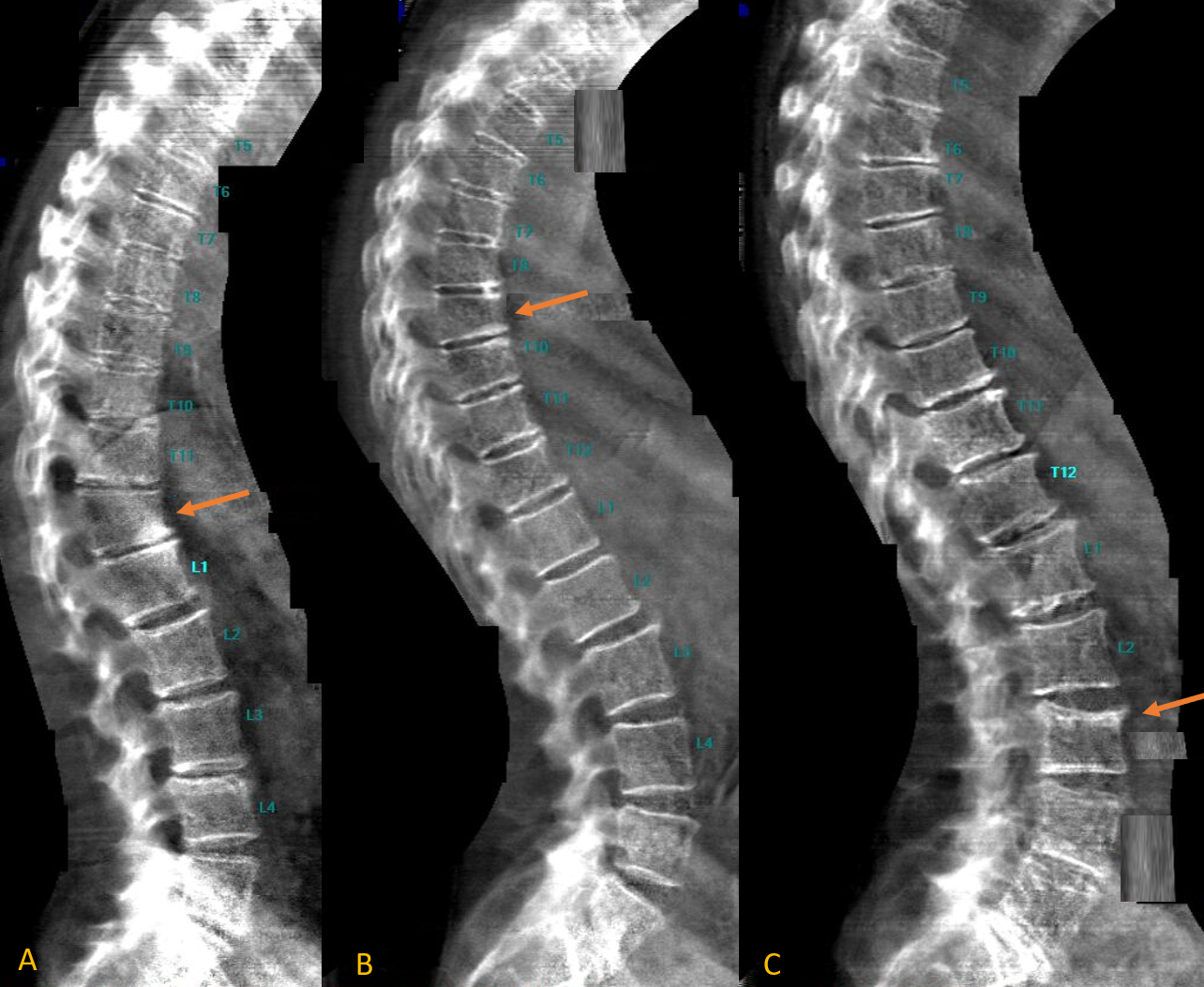


***Suppl Fig-2*.** Dual-energy X-ray absorptiometry images (VFA: vertebral fracture assessment) show osteoporotic-like vertebral fractures (OLVF, T12 in **A**, T9 in **B**, and L3 in **C**, arrows).


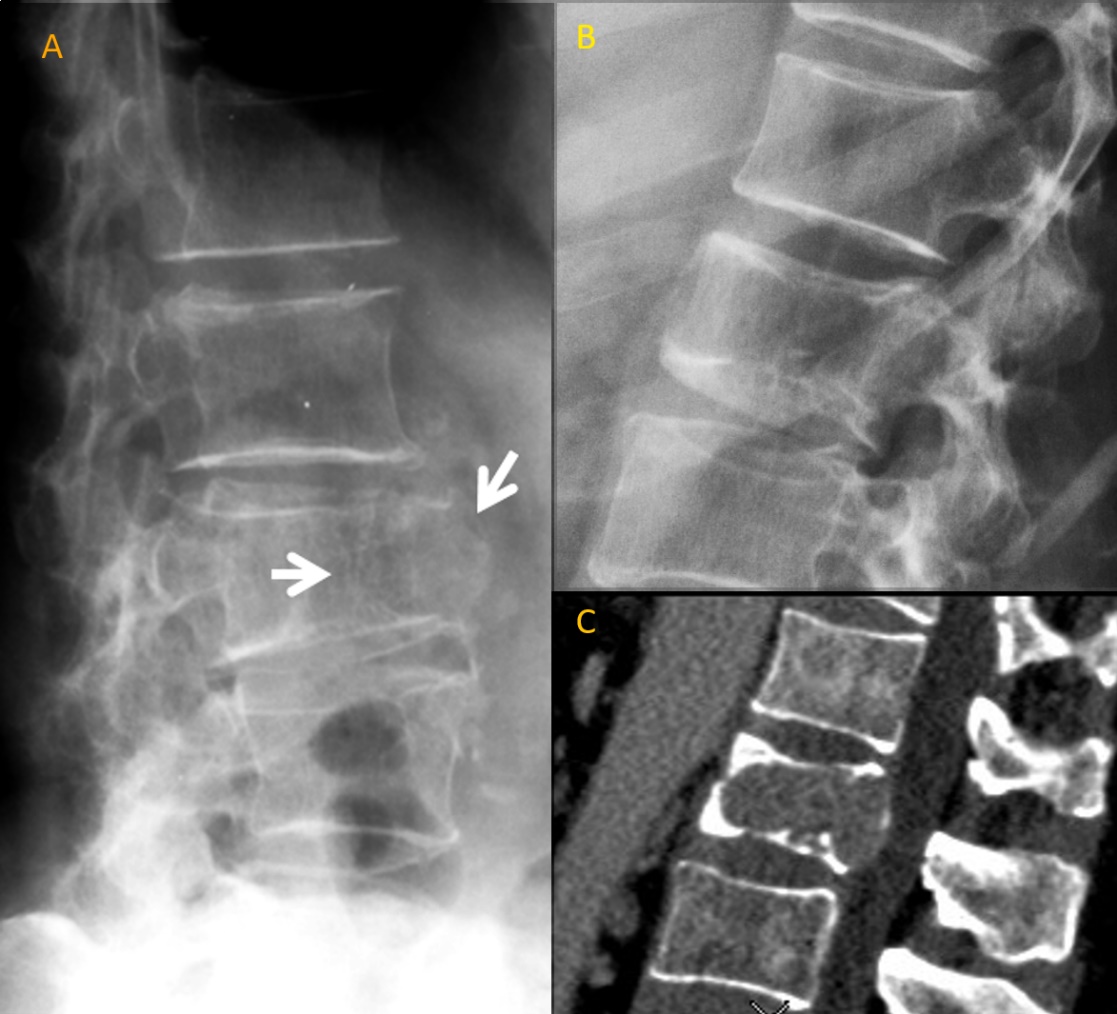


***Suppl Fig-3****.* Two cases of oncological vertebral deformities which can be easily differentiated from OLVF (osteoporotic-like vertebral fracture). **(A):** Radiograph of a 68-year-old woman shows osteolytic pathological fracture of L3 due to malignant tumor (arrows). The lesion shows loss of continuity of the bone cortex and trabecular network in the anterior aspect of the vertebral body with destructive and expansive features. The anterosuperior aspect of L4 also shows destructive features. Radiograph **(B)** and CT **(C):** a case of vertebral metastasis of lung carcinoma in L2. L2 shows loss of height and both superior and inferior endplate depression. The inferior endplate demonstrates an irregular shape which differs from osteoporotic depression. The convexity of the posterior vertebral wall, as shown on CT, is characteristic of pathologic vertebral fractures. *Reproduced with permission from* *Wang et al. Quant Imaging Med Surg. 2017;7:555–591.*


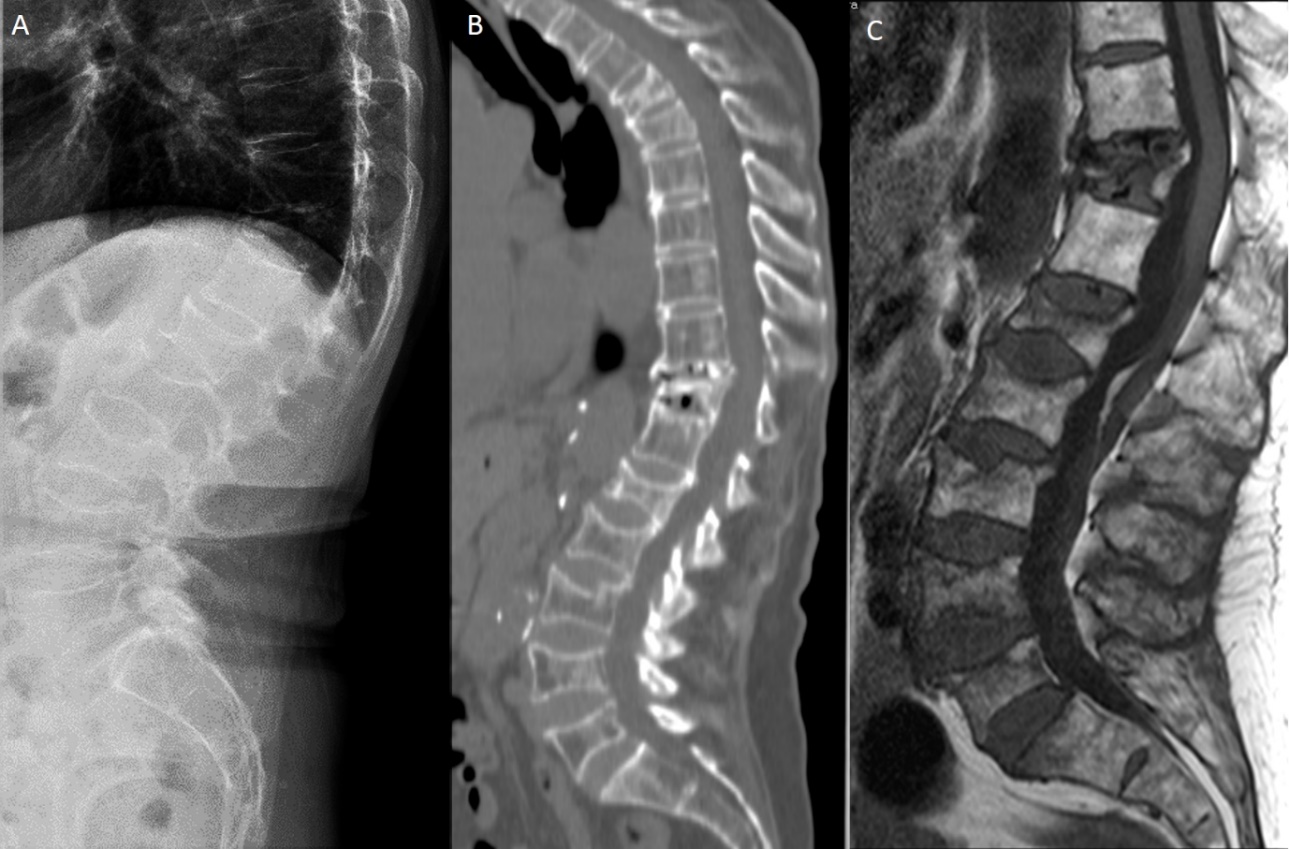


***Suppl Fig-4*.** Radiograph **(A),** CT **(B)** and sagittal T1-weighted MRI **(C)** show a case of multiple myeloma. Vertebral fractures of various severities are seen on radiographs. On radiographs no definite differentiation can be made with osteoporotic fracture. *Reproduced with permission from* *Wang et al. Quant Imaging Med Surg. 2017;7:555–591*.


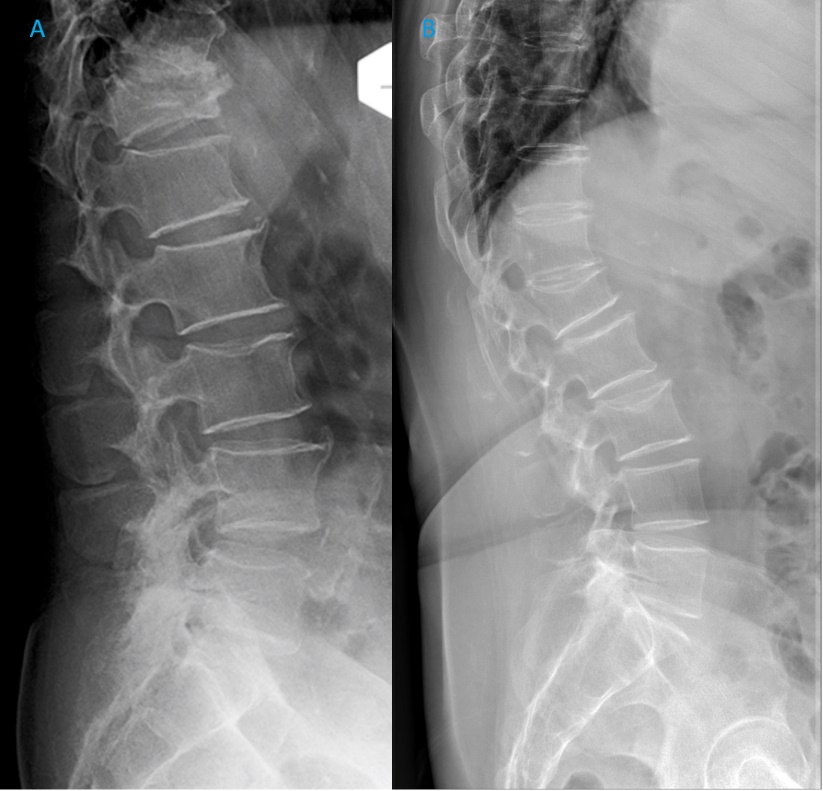


***Suppl Fig-5*.** Endplate depression with maintained vertebral height. **(A):** lumbar radiograph of an 85-year-old male. T12 is collapsed. The superior endplate of L3 demonstrates slight depression with well-maintained anterior vertebral height. **(B):** lumbar radiograph of a 74-year-old female. Severe grade osteoporotic deformity of L1. The superior endplate of L3 demonstrates slight depression with well-maintained anterior vertebral height. *Reproduced with permission from Wáng. Quant Imaging Med Surg. 2023 ;13:1264-1285*


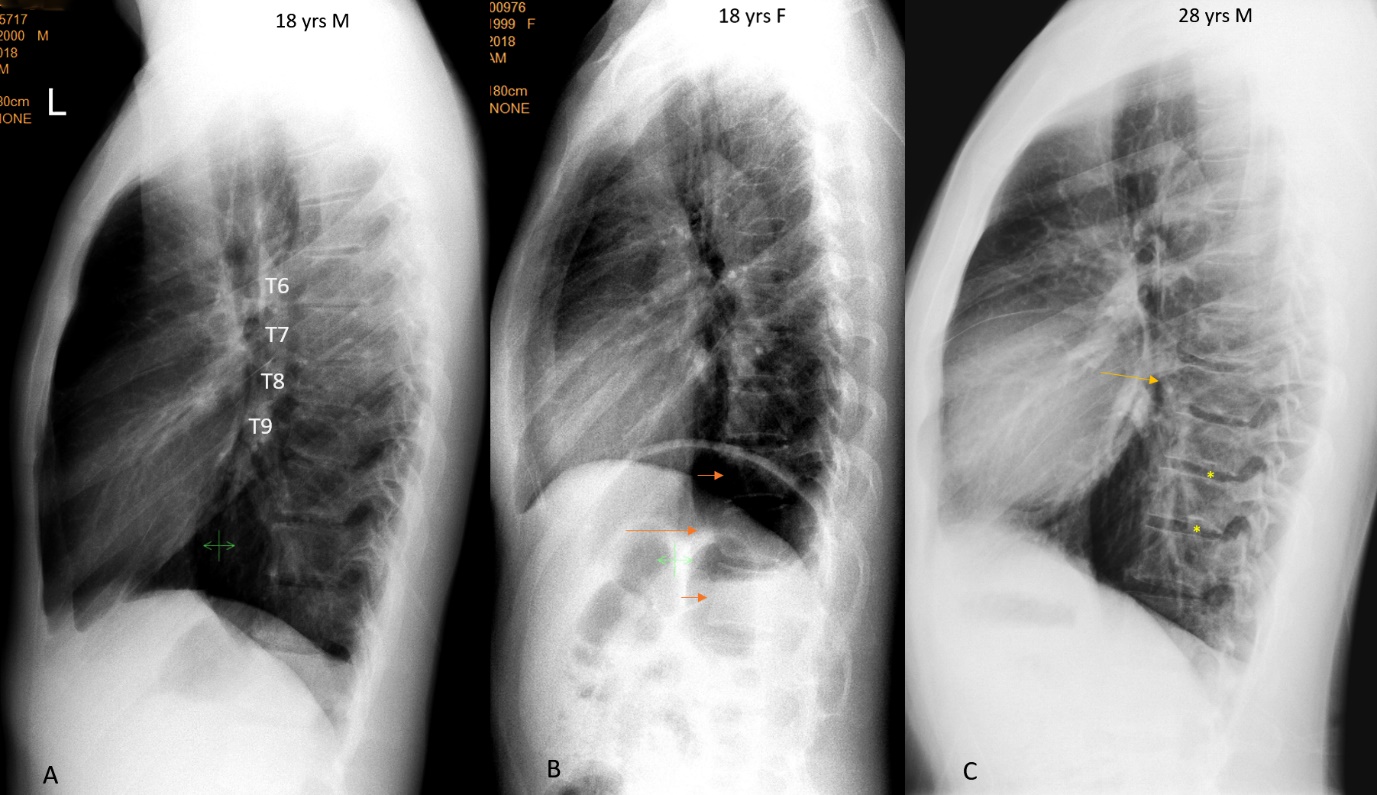


***Suppl Fig-6*.** Three cases of congenital short vertebra. **(A):** lateral radiograph of an 18-year-old man. T8 has a reduced height, and the size of T7 is larger than usual while the size of T9 is smaller than usual. T10 has a longer than usual anterior length. The T6/T7 and T7/T8 intervertebral disc spaces are narrowed. **(B):** lateral radiograph of an 18-year-old woman. T11 has a reduced height (long arrow). The anterosuperior and anteroinferior corners are smooth. T10 and T12 (short arrows) demonstrate compensated overgrow. **(C):** Lateral radiograph of a 28-year-old man. T8 has a reduced height (arrow), and the aspect border appears to be irregular. The inferior endplates of T9 and T10 both have a notch (asterisk), suggesting developmental Schmorl’s nodes. *Reproduced with permission from Ma and Wáng. J Thorac Dis. 2022;14:4685-4698, and Wáng. Quant Imaging Med Surg. 2023 ;13:1264-1285*


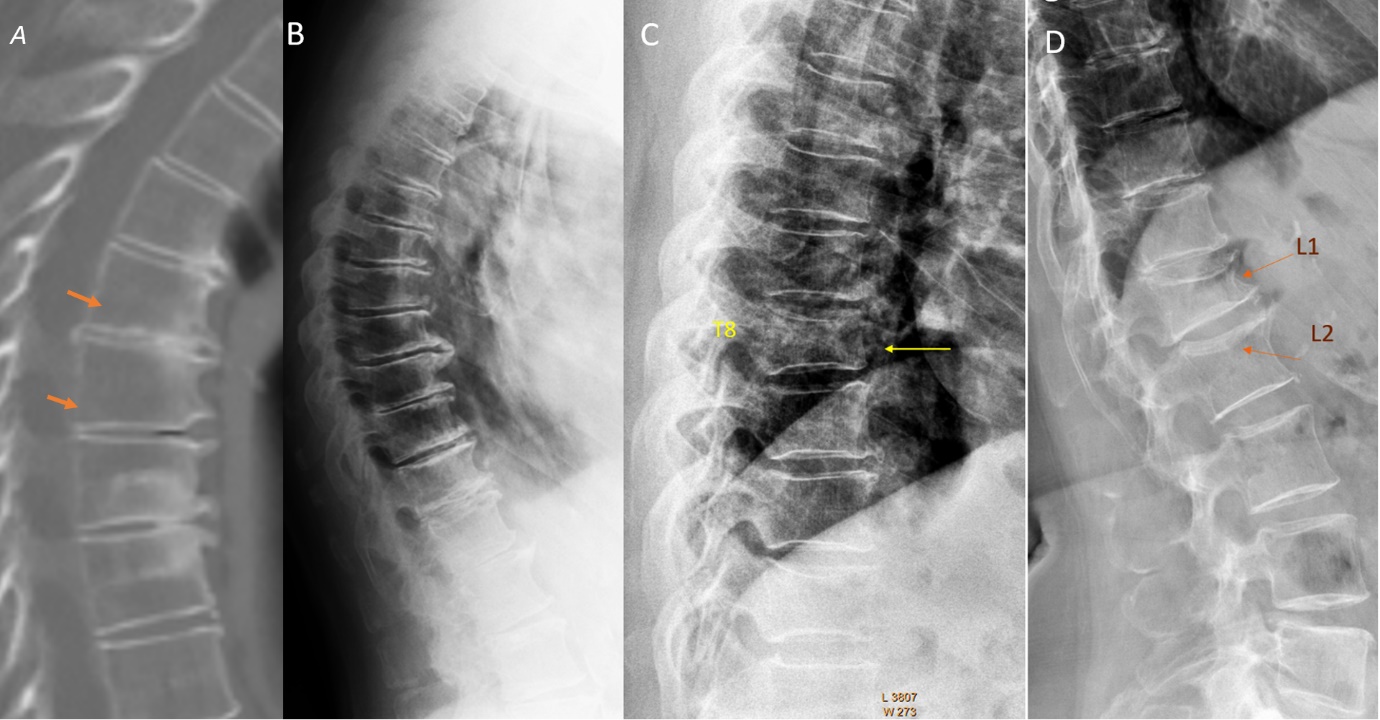


***Suppl Fig-7*.** Example of osteoarthritic (OA) wedging, acquired short vertebrae (SVa), and OLVF. **(A)** OA wedging. Sagittal CT reconstruction shows vertebrae with osteoarthritic wedging, marginal osteophytes, and disc space narrowing; two adjacent vertebrae (arrows) have anterior wedging with minimal/mild height loss**. (B)** Lateral radiograph of an elderly woman. In the mid-thoracic region, there is anterior wedging of multiple vertebrae with similar appearance, disc space narrowing, and osteophytes, without endplate depressions. There are features of both OA wedging and SVa in this case. (**C**) Spine from a lateral chest radiograph of an elderly woman. T8 shows deformity consistent with OLVF (arrow). (**D**) Lateral radiograph of an elderly woman. L1 shows deformity consistent with OLVF (arrow), while L2 shows superior endplate depression (i.e., endplate fracture) (arrow). OLVF, osteoporotic-like vertebral fracture. *Reproduced with permission from Wáng et al. Aging Clin Exp Res. 2023;35:2583-2591, and Wáng. Quant Imaging Med Surg . 2023;13:1264-1285*


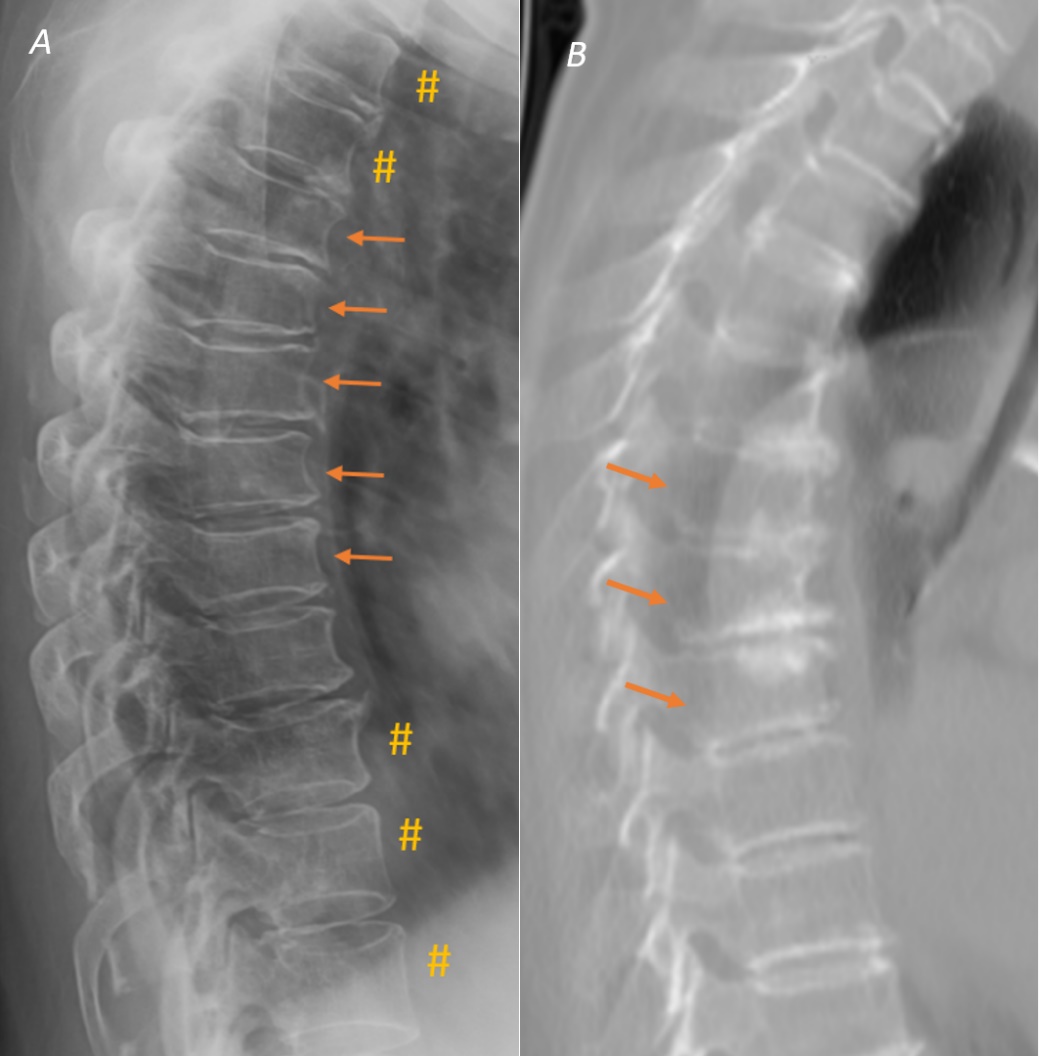


***Suppl Fig-8*.** Example of acquired short vertebrae (SVa) and osteoarthritic (OA) wedging. **(A):** Lateral thoracic spine radiograph showing SVa (arrows show multiple SVa, #: vertebrae assumed of normal shape). **(B):** OA wedging. Sagittal CT reconstruction shows vertebrae with OA wedging demonstrating marginal osteophytes and disc space narrowing. The involved adjacent vertebrae (arrows) demonstrate anterior wedging with minimal/mild height loss. In **(B),** the increased density of the involved endplates suggests regenerative inflammatory changes, without apparent endplate fractures. *Reproduced with permission from Wáng et al. Aging Clin Exp Res. 2023;35:2583-2591.*


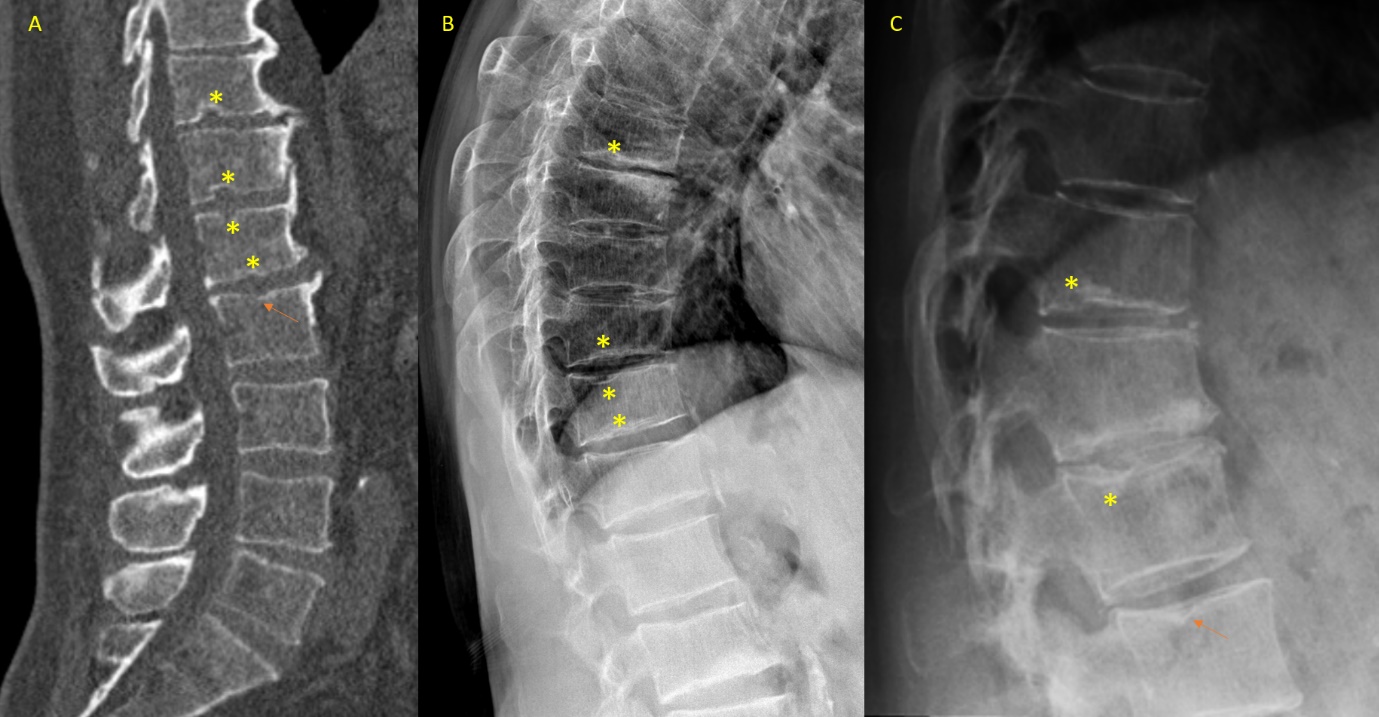


***Suppl Fig-9*.** Image examples **(A, B, C)** of Schmorl’s nodes of developmental cause (SNd). (**A**) Reconstructed CT image. (**B**) Lateral radiograph. Multiple short vertebrae are noted in **B**. (**C**) Lateral radiograph. SNd are marked with asterisks and arrows indicate possible SNd. *Reproduced with permission from Wáng. Quant Imaging Med Surg 2023;13:4044-4049.*


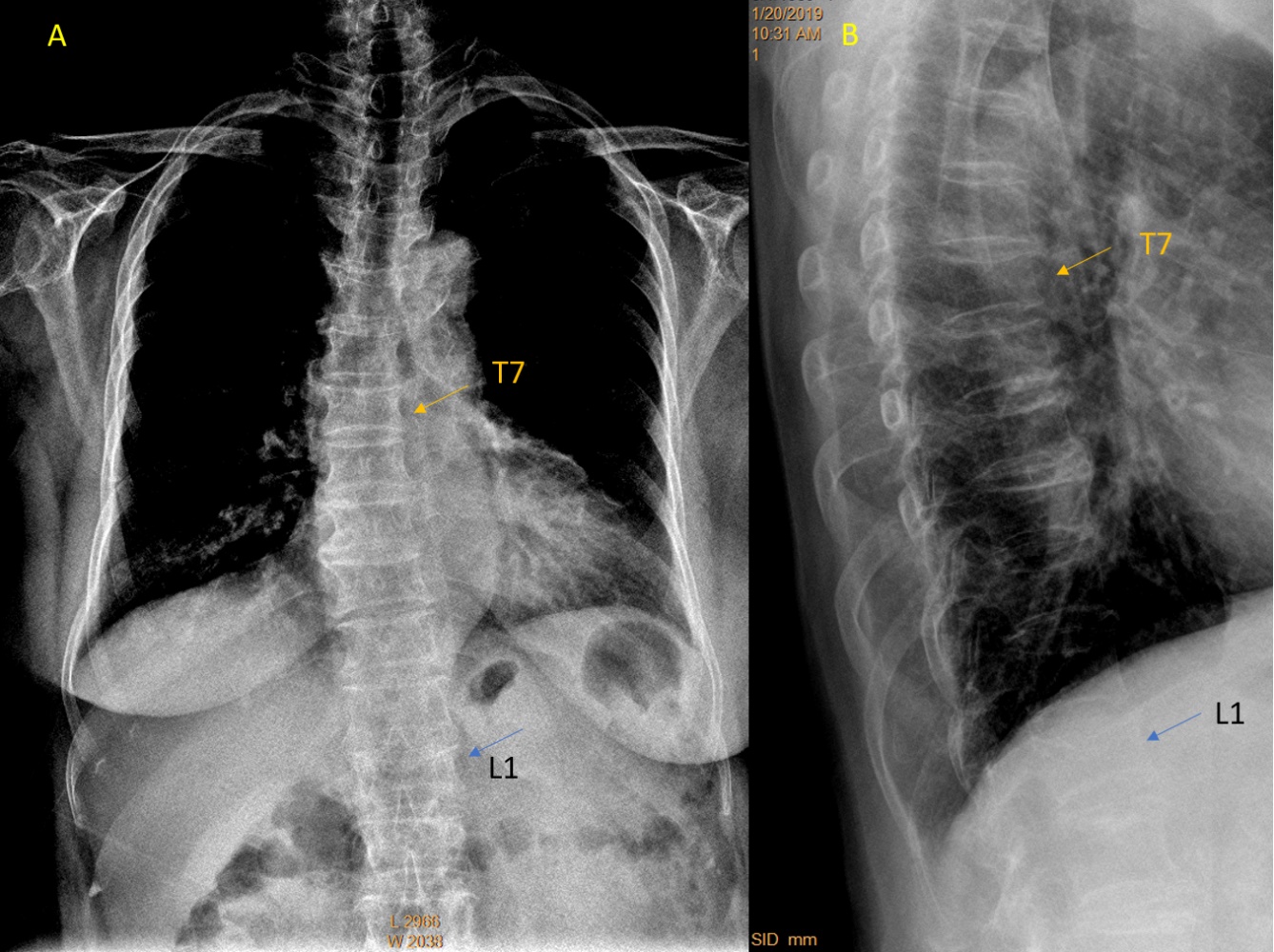


***Suppl Fig-10*.** Chest radiographs of an 84-year-old female. **(A):** Frontal view radiograph shows OVLF at L1, which is apparent when comparing with the normal appearing morphology of T12 and L2. **(B):** The OVLF at L1 is confirmed on lateral radiograph. T7 has a mild grade OVLF which cannot be readily detected on frontal review radiograph. OLVF, osteoporotic-like vertebral fracture. *Reproduced with permission from Wáng et al. Quant Imaging Med Surg. 2021;11:423-442.*


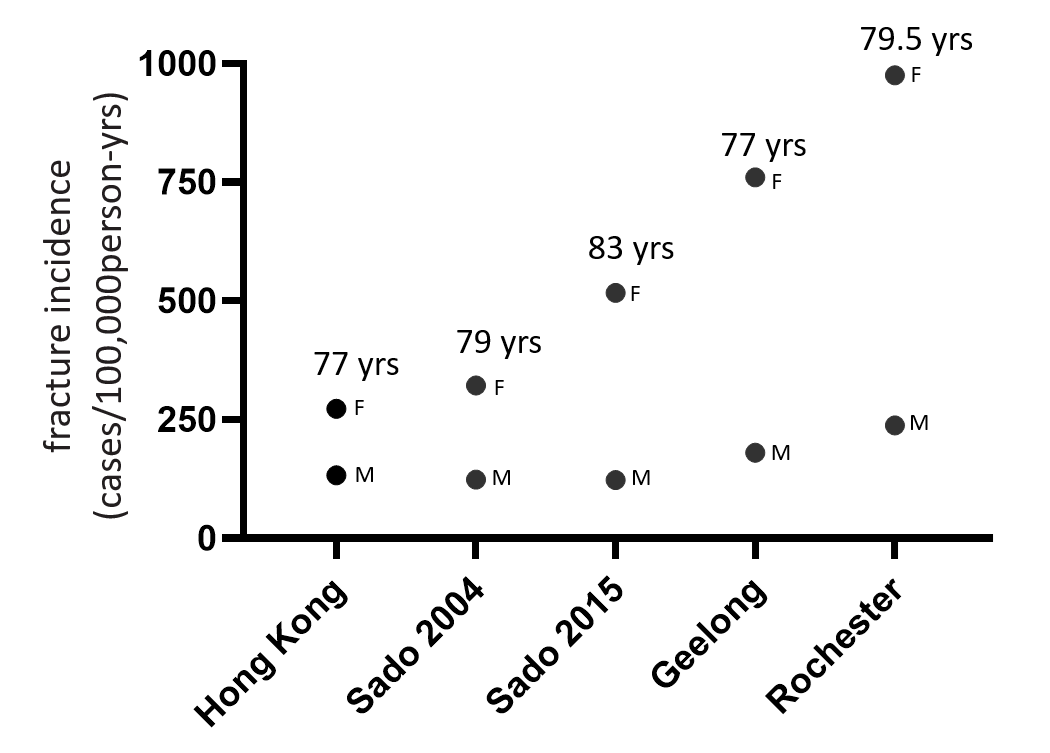


***Suppl Fig-11*:** Incidences of clinical osteoporotic vertebral fracture in older men are mostly less than half of the incidences of clinical osteoporotic vertebral fracture in older women. Data are from Wáng et al. Quant Imaging Med Surg. 2022;12:2090-2105 (MrOs(Hong Kong) and MsOS (Hong Kong) studies); Sakuma et al, J Bone Miner Metab 2008;26:373–378 (Japan Sado 2004), Imai et al, J Bone Miner Metab 2019; 37:484–490 (Japan Sado 2015); Sanders et al. Osteoporos Int 1999;10:240-7 (Geelong study); Cooper et al. J Bone Miner Res 1992;7:221-7 (Rochester study). The mean ages (yrs: years) during the follow-up period for each study are noted. M: men’s data, F: women’s data.
